# Ocular Manifestations of Acute Secondary Angle Closure Associated With Lens Subluxation

**DOI:** 10.3389/fmed.2021.738745

**Published:** 2022-01-14

**Authors:** Qinghe Jing, Tianhui Chen, Zexu Chen, Lina Lan, Chen Zhao, Yongxiang Jiang

**Affiliations:** ^1^Department of Ophthalmology and Vision Science, Eye & Ear Nose and Throat (ENT) Hospital of Fudan University, Shanghai, China; ^2^NHC Key Laboratory of Myopia (Fudan University), Key Laboratory of Myopia, Chinese Academy of Medical Sciences, and Key Laboratory of Visual Impairment and Restoration of Shanghai, Shanghai, China

**Keywords:** acute angle closure, lens subluxation, anterior chamber depth, inter-eye ACD difference, glaucoma

## Abstract

**Purpose:**

To evaluate the clinical characteristics and ocular features of patients with acute secondary angle closure, associated with lens subluxation (ASAC-LS).

**Methods:**

We performed a retrospective study at the EENT Hospital of Fudan University, Shanghai, China. A total of 41 affected eyes from 41 patients were enrolled in this study. Furthermore, 20 affected eyes were part of the ASAC-LS cohort and 21 affected eyes were included in the acute primary angle closure (APAC) cohort. The best-corrected visual acuity (BCVA), intraocular pressure (IOP), axial length (AL), minimum corneal curvature (K1), maximum corneal curvature (K2), and anterior chamber depth (ACD) were measured and compared between the 2 cohorts. In addition, inter-eye (intraindividual) comparison was performed.

**Results:**

The ASAC-LS cohort exhibited younger ages, more frequent trauma history (35%), lower IOP (27.43 ± 13.86 mmHg vs. 41.27 ± 10.36 mmHg), longer AL (23.96 ± 2.60 vs. 22.49 ± 0.77 mm), shallower ACD (1.28 ± 0.38 vs. 1.58 ± 0.23 mm), and bigger ACD differences (0.99 ± 0.52 vs. 0.15 ± 0.19 mm), as compared with the APAC cohort (all *p* < 0.05). Moreover, eyes from the lens subluxation cohort experienced worse BCVA, higher IOP, and shallower ACD than their matched unaffected eyes (all *p* < 0.05). Although longer AL, shallower ACD, and bigger ACD differences were strongly correlated with lens subluxation in a univariate logistic regression analysis, only the ACD difference remained significant in the multivariate model (*p* = 0.004, *OR* = 1,510.50). Additionally, according to the receiver operating characteristic (ROC) curve analysis, both ACD and ACD differences had greater value in the differential diagnosis of ASAC-LS and APAC, with a cut-off value of 1.4 and 0.63 mm, respectively.

**Conclusions:**

Shallower ACD and larger ACD differences provide the promising diagnostic potential for patients with ASAC-LS.

## Introduction

Lens subluxation has multi-factorial etiology, including congenital or developmental conditions (such as Marfan syndrome, Weill–Marchesani syndrome, and homocystinuria), comorbidities of eye diseases, associated with zonular disease (such as high myopia, retinitis pigmentosa, pseudoexfoliation syndrome, and uveitis), and a history of ocular blunt trauma or previous intraocular surgery (1–7). This condition can sometimes induce acute secondary angle closure (ASAC) that presents with non-specific signs and symptoms, similar to acute primary angle closure (APAC), such as blurred vision, elevated intraocular pressure (IOP), shallow anterior chamber, severe ocular pain, headache, nausea, and vomiting. According to earlier studies, about 4.1–21% of ASAC cases, associated with lens subluxation (ASAC-LS) were misdiagnosed as APAC (8–11). Due to a wide range of causes for angle closure, the management and operation protocols for these patients can be vastly different. For instance, in patients with APAC, pupil size must be regulated using a miotic agent to remove the pupillary block. However, in patients with ASAC-LS, a mydriatic agent must be used instead to enlarge the anterior chamber and open the angle. Therefore, it is important to differentiate the cause of angle closure during a clinical examination. In this study, we delineated the clinical and ocular characteristics of patients with ASAC-LS and APAC, which may benefit the future diagnosis and differential diagnosis of these patients.

## Methods

A retrospective study was conducted at the Eye and ENT Hospital of Fudan University, Shanghai, China. This study was approved by the Human Research Ethics Committee of the Eye and ENT Hospital of Fudan University (no. 2020103) and was performed with adherence to the tenets of the Declaration of Helsinki. Written informed consent was obtained from all patients.

The medical records of patients, with ASAC-LS and APAC diagnoses, between June 2016 and July 2019, were reviewed. The criteria for ASAC-LS, used in this study, were as follows: (1) decreased vision, sudden pain in the eye, nausea, and vomiting; (2) silt-lamp microscope revealing shallower ACD and/or uneven ACD among the quadrants, iridodonesis and/or phacodonesis, decentration of the nucleus with or without vitreous in the anterior chamber; (3) ultrasound biomicroscopy (UBM) showing shallow central and peripheral anterior chamber depth (ACD), asymmetrical ACD in the same eye, tilting of the lens, and/or asymmetrical iris configuration. Lens subluxation was reconfirmed during the subsequent surgery. APAC was diagnosed with the following symptoms: (1) presence of at least one of the following symptoms: ocular or periocular pain, headache, nausea and/or vomiting, an antecedent history of intermittent blurring of vision and haloes; (2) elevated IOP, closed angle; (3) slit-lamp microscope finding of at least three of the followings: ciliary injection or mixed injection, corneal edema, mid-dilated pupil, presence of glaucoma flecks, and shallow peripheral anterior chamber depth. Patients with a history of corneal disease, glaucoma, uveitis, corneal surgery, and intraocular surgery were excluded from the study.

The patients' standard demographic and clinical characteristics of both eyes were retrieved from their medical records, namely, age, gender, laterality of affected eye, cause of lens subluxation, antiglaucoma surgery [laser peripheral iridectomy (LPI) or LPI and Argon laser peripheral iridoplasty (ALPI)], best-corrected visual acuity (BCVA), and intraocular pressure (IOP). In addition, data from the IOLMaster (Cal Zeiss Meditec, Jena, Germany), such as axial length (AL), minimum corneal curvature (K1), and maximum corneal curvature (K2) were collected. The anterior chamber depth (ACD), chamber angle, and zonular status were detected using UBM (MD-300L, MEDA, Tianjin, China). Snellen visual acuity measurements were converted to logarithm of the minimum angle of resolution (logMAR) equivalents for data analysis. Finally, the ACD difference was calculated by ACD in the contralateral eye minus ACD in the affected eye.

All data analyses were performed with SPSS version 18 (IBM Corp., Armonk, NY, USA). Continuous variables are presented as mean ± SD. Categorical variables were compared with the chi-square test or Fisher's exact test. Continuous variables were compared with independent-sample *t*-test or Mann–Whitney *U*-test, depending on data distribution. Logistic regression analysis was used to identify the factors associated with ASAC-LS. For diagnostic assessment, the values of ACD in the affected eye and the inter-eye ACD difference were calculated from the area under the receiver operating characteristic (AUROC) curves and were used to delineate between the ASAC-LS and APAC cohorts. A *p* < 0.05 was considered a significant difference.

## Results

A total of 41 affected eyes (from 41 participants) were included in this study; 20 of the affected eyes belonged to the ASAC-LS cohort and 21 affected eyes belonged to the APAC cohort. Generally, the patients with lens subluxation were younger than the patients with APAC, with a mean age of 61.05 ± 11.28 and 66.71 ± 9.72 years, respectively (*p* = 0.04). Despite a female preponderance in each group, the difference in sex ratio between the groups was not statistically significant (*p* = 0.655). Among the lens subluxation cohort, 7 cases (35%) had a history of eye or head trauma, which was significantly high compared with the control cohort (*p* = 0.01). The demographic and past medical histories of the ASAC-LS and APAC cohorts are summarized in Table 1. Only 3 patients (15%) in the study group had lens subluxation in both eyes. Among them, 1 was associated with retinitis pigmentosa (RP), 1 with familial lens subluxation, and 1 with spontaneous lens subluxation. These patients were excluded from the ACD difference analysis.

**Table 1:** The demographic and past histories of the ASAC-LS and APAC cohorts.

**Variables**	**APAC**	**ASAC-LS**	**P Value**
No. of subjects	21	20	
Age (y)^a^	66.71 ± 9.72	61.05 ± 11.28	0.04^*^
Sex (Male:Female)^b^	5:16	6:14	0.655
Eyes (Right:Left)^b^	11:10	9:11	0.636
Surgery History^b^	8	3	0.095
Trauma History^c^	0	7	0.01^*^

a *Mann-Whitney U test*;

b *Chi-squared Test*;

c *Fisher Exact Test*;

* *Value with statistical significance*.

*APAC, acute primary angle closure; ASAC-LS, acute secondary angle closure associated with lens subluxation. Surgery history contains laser peripheral iridectomy (LPI) or LPI and Argon laser peripheral iridoplasty (ALPI)*.

Table 2 presents the ocular biometric data from both eyes of the ASAC-LS and APAC cohorts. The IOP, AL, and ACD in the affected eye along with the BCVA, AL, and ACD in the contralateral eye and the ACD difference between the affected and contralateral eyes were remarkably different between the two cohorts. The IOP of APAC was 41.27 ± 10.36 mmHg, whereas that of the ASAC-LS cohort was 27.43 ± 13.86 mmHg (*p* = 0.001). The AL of the affected and unaffected eyes within the study groups were significantly longer than those of the control group (*p* = 0.001 and *p* < 0.001, respectively). Only 2 patients exhibited AL ≥ 30 mm in the lens subluxation cohort, whereas the rest of the patients had AL < 26 mm. ACD was shallower in the affected eyes of the lens subluxation cohort (range: 0.32–2.03 mm; *p* = 0.004), and deeper in the contralateral eyes of the same study group (range: 1.03–4.31 mm; *p* < 0.001). In addition, the ACD difference was bigger in the ASAC-LS cohort than those in the APAC cohort (*p* < 0.001). The BCVA in the unaffected eye of the lens subluxation cohort was better than the APAC cohort (*p* = 0.032). In addition, the BCVA, K1, and K2 in the affected eye, and the IOP, K1, and K2 in the unaffected eye showed no statistically significant difference between the two cohorts.

**Table 2:** The ocular biometric data from both eyes of the ASAC-LS and APAC cohorts.

**Variables**		**APAC**	**ASAC-LS**	***P* Value**
Affected eye	BCVA ^a^	1.38 ± 0.99	1.30 ± 1.17	0.763
	IOP (mmHg)^b^	41.27 ± 10.36	27.43 ± 13.86	0.001^*^
	AL (mm)^a^	22.49 ± 0.77	23.96 ± 2.60	0.001^*^
	K1 (D)^c^	44.25 ± 1.56	43.79 ± 1.14	0.292
	K2 (D)^a^	45.23 ± 1.62	44.74 ± 1.24	0.411
	ACD (mm)^c^	1.58 ± 0.23	1.28 ± 0.38	0.004^*^
Fellow eye	BCVA ^a^	0.80 ± 1.42	0.23 ± 0.28	0.032^*^
	IOP (mmHg)^a^	15.06 ± 6.57	16.34 ± 9.45	0.583
	AL (mm)^c^	22.37 ± 0.70	23.59 ± 1.28	0.000^*^
	K1 (D)^c^	43.80 ± 1.76	43.68 ± 1.69	0.824
	K2 (D)^c^	45.30 ± 1.66	44.64 ± 1.51	0.193
	ACD (mm)^c^	1.72 ± 0.27	2.21 ± 0.44	0.000^*^
ACD difference (mm)^b^		0.15 ± 0.19	0.99 ± 0.52	0.000^*^

a *Mann-Whitney U-test*;

b *Corrected T Test*;

c *T Test*;

* *Value with statistical significance*.

*APAC, acute primary angle closure; ASAC-LS, acute secondary angle closure associated with lens subluxation; BCVA, best corrected visual acuity; IOL, intraocular pressure; AL, axial length; D, diopter; K1, minimum corneal curvature; K2, maximum corneal curvature; ACD, anterior chamber depth; ACD difference, ACD (fellow eye) – ACD (affected eye)*.

**Figure 1:**
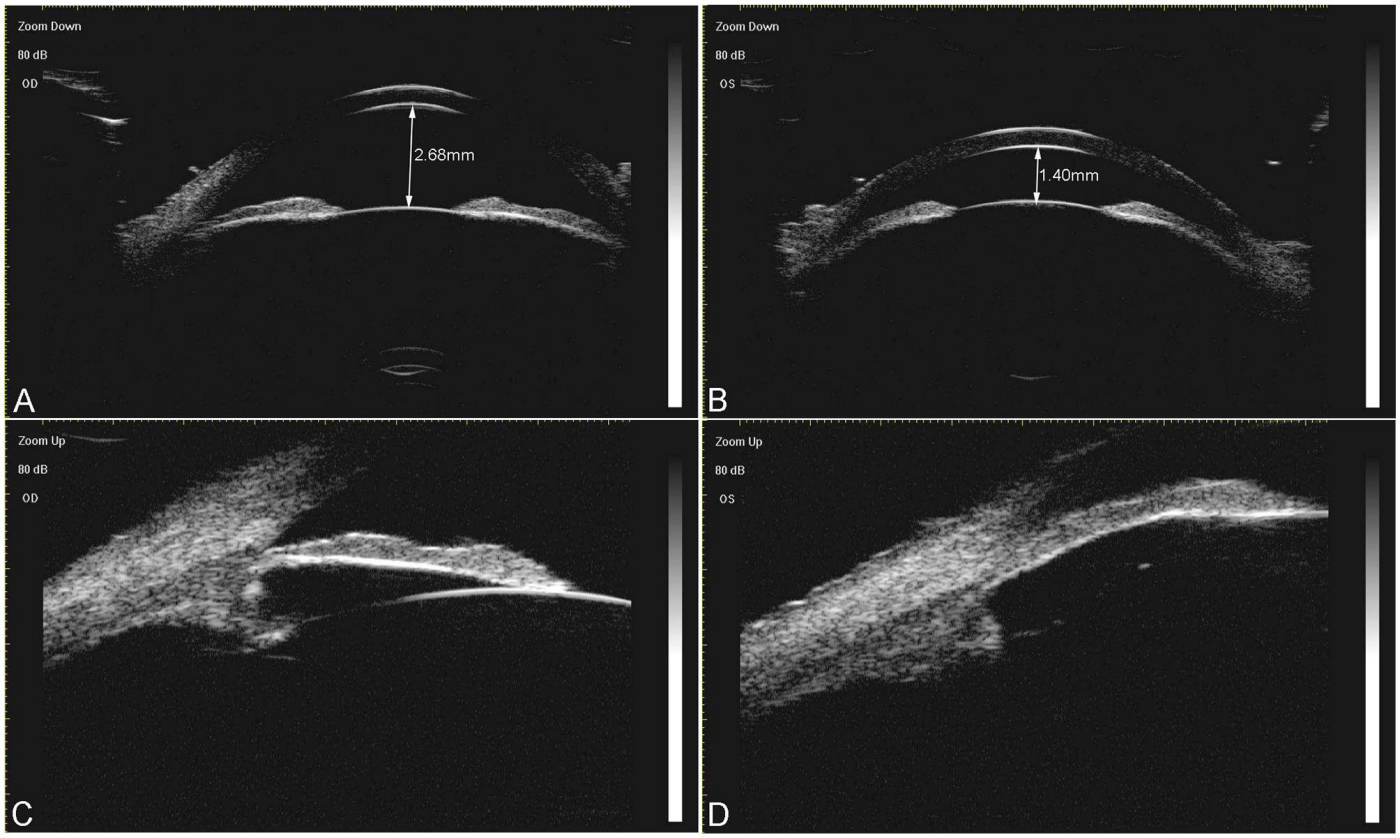
A representative case of acute secondary angle closure associated with the lens subluxation (ASAC-LS) group. A 52-year-old woman had a history of acute angle closure attack in her left eye. The ultrasound biomicroscopy (UBM) images prior to surgery revealed an obvious shallow central anterior chamber depth in her left eye [**(B)**, 1.40 mm), compared with the right eye [**(A)**, 2.68 mm]. **(C)** Illustrates that the angle was open in the right eye. **(D)** Depicts that the angle was closed in the left eye, the zonule was sparse in each quadrant, and the lens moved forward slightly.

Table 3 illustrates inter-eye comparisons between the affected eyes and their corresponding unaffected eyes of the ASAC-LS and APAC cohorts. The BCVA of the affected eyes was worse than the unaffected eyes in both cohorts (all *p* < 0.01), and the IOP of the affected eyes was higher than the unaffected eyes in both cohorts (all *p* < 0.01). In addition, the ACD of the affected eyes was significantly shallower than the unaffected eyes in the lens subluxation cohort (*p* < 0.001). Figure 1 illustrates a representative case from the ASAC-LS group which exhibited a great difference in ACD in the center of each eye. Moreover, the same tendency was observed in the APAC cohort, however, the result was not statistically significant (*p* = 0.066). In addition, the inter-eye comparisons revealed no significant differences in the field of AL, K1, and K2 in both cohorts.

**Table 3:** The intereye comparison of subjects in the acute primary angle closure cohort and acute secondary angle closure cohort, associated with lens subluxation.

**Variables**		**Affected eye**	**Fellow eye**	***P* Value**
APAC	BCVA ^a^	1.38 ± 0.99	0.80 ± 1.42	0.002^*^
	IOP (mmHg)^a^	41.27 ± 10.36	15.06 ± 6.57	0.000^*^
	AL (mm)^b^	22.49 ± 0.77	22.37 ± 0.70	0.586
	K1 (D)^b^	44.25 ± 1.56	43.80 ± 1.76	0.380
	K2 (D)^a^	45.23 ± 1.62	45.30 ± 1.66	0.930
	ACD (mm)^b^	1.58 ± 0.23	1.72 ± 0.27	0.066
ASAC-LS	BCVA^a^	1.30 ± 1.17	0.23 ± 0.28	0.000^*^
	IOP (mmHg)^a^	27.43 ± 13.86	16.34 ± 9.45	0.007^*^
	AL (mm)^a^	23.96 ± 2.60	23.59 ± 1.28	0.586
	K1 (D)^b^	43.79 ± 1.14	43.68 ± 1.69	0.380
	K2 (D)^b^	44.74 ± 1.24	44.64 ± 1.51	0.930
	ACD (mm) ^b^	1.28 ± 0.38	2.21 ± 0.44	0.000^*^

a *Mann-Whitney U test*;

b *T Test*;

* *Value with statistical significance*.

*APAC, acute primary angle closure; ASAC-LS, acute secondary angle closure associated with lens subluxation; BCVA, best corrected visual acuity; IOL, intraocular pressure; AL, axial length; D, diopter; K1, minimum corneal curvature; K2, maximum corneal curvature; ACD, anterior chamber depth*.

Table 4 summarizes the results of the logistic regression analysis that identified the risk factors for lens subluxation. According to the univariate model, AL, ACD, and ACD differences exhibited a notable correlation with lens subluxation (all *p* < 0.05). However, only the ACD difference remained significant in the multivariate model. In fact, a 1 mm increase in ACD difference was associated with 1,510.50 times greater risk of lens subluxation.

**Table 4:** Univariate and multivariate logistic regression analysis for the identification of risk factors of acute secondary angle closure, associated with lens subluxation.

**Variables**	**Univariate**		**Multivariate**	
	**OR**	***P* Value**	**OR (95%CI)**	***P* value**
Age (y)	0.947	0.101		
Sex	0.729	0.655		
Trauma History	2.61^*^10^9^	0.999		
AL (mm)	3.769	0.017^*^		0.51
ACD (mm)	0.033	0.01^*^		0.53
ACD Difference (mm)	1510.50	0.004^*^	1510.50(10.50-217237.26)	0.004^*^

* *Value with statistical significance*.

*AL, axial length; ACD, anterior chamber depth; ACD difference, ACD (fellow eye) – ACD (affected eye); OR, odd ratio; CI, confidence interval*.

*Factors with P < 0.10 in the univariate model (AL, ACD, ACD Difference) were applied to the multivariate model*.

**Figure 2:**
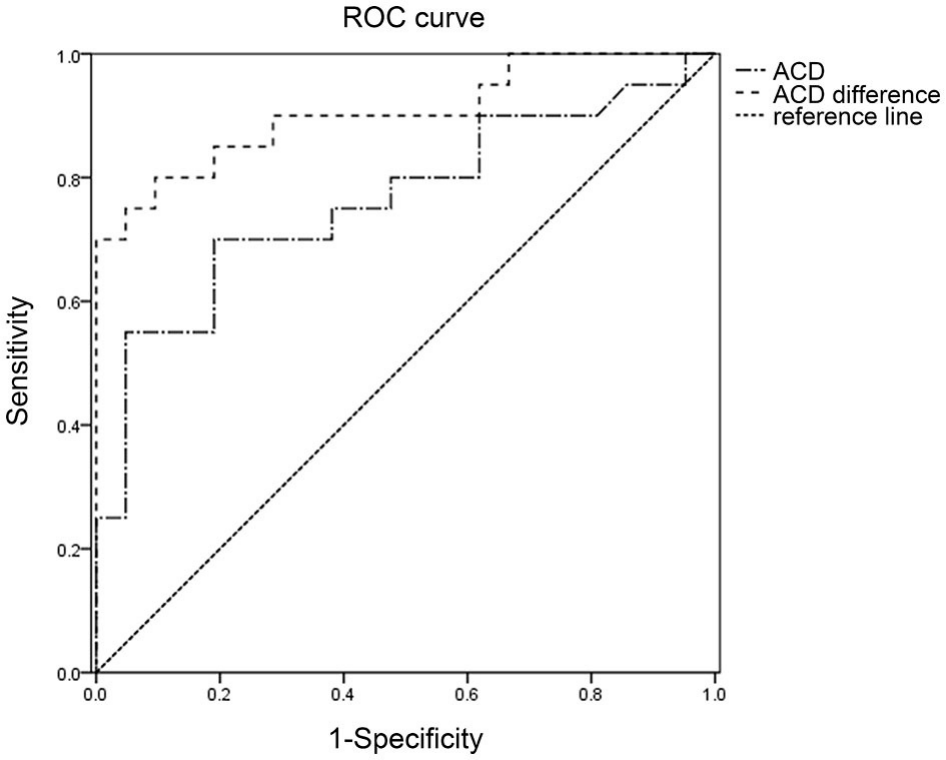
The receiver operating characteristic (ROC) curve for ACD and ACD difference, in relation to the diagnosis of ASAC-LS. ROC, Receiver operating characteristic; ACD, anterior chamber depth; ASAC-LS, acute secondary angle closure associated with lens subluxation.

The receiver operating characteristic (ROC) curve was used to determine the potential diagnostic value of ACD and ACD difference between the lens subluxation and APAC cohorts, as shown in Figure 2. The AUROC for ACD was 0.763 and the ACD difference was 0.966. The value of ACD at 1.4 mm was found to be the optimal cut-off point for the ASAC-LS and APAC cohorts, with a sensitivity of 70.0% and a specificity of 81.0% (*p* < 0.001). Moreover, the ACD difference at 0.63 mm had a specificity of 100.0% and a sensitivity of 82.4% (*p* < 0.001).

## Discussion

Lens subluxation is a multifactorial condition that involves a common malpositioning of the lens, likely due to the partial zonular dehiscence or zonular laxity/weakness (12). Among the 20 lens subluxation cases examined in this study, 7 were caused by trauma, 2 cases by high myopia, 1 case by RP, 1 case had familial lens subluxation, 1 case had spontaneous lens subluxation, and the remaining cases had unknown causes. Determining the etiology of this disease can aid in the accurate diagnosis and proper management of personalized therapy. So, it is crucial to obtain a detailed medical history and examine patients thoroughly to significantly improve the diagnostic accuracy rate of lens subluxation.

The common manifestations of lens subluxation are progressive refractive modifications (myopia and astigmatism) with decreased vision (amblyopia in children), glaucoma (through different mechanisms), and in some cases retinal detachment (13). The ocular signs of lens subluxation include abnormal ACD (shallower ACD, deeper ACD, uneven ACD among the quadrants, and great difference in ACD in the center of each eye), iridodonesis, phacodonesis, anterior displacement of the iris lens diaphragm, decentration of the nucleus, visible lens equator margin through the dilated pupil, and vitreous in the anterior chamber (8, 10).

With the development of technology, multiple anterior segment imaging instruments can be used to objectively and accurately visualize and evaluate anterior segment parameters, such as the Scheimpflug imaging-based system (Pentacam), anterior segment optical coherence tomography (AS-OCT), and UBM (14). Although Pentacam and AS-OCT can acquire images and data relatively quickly and without corneal contact, they are still affected by the size of the pupil and the clarity of the refractive media. UBM is a high-frequency, high-resolution imaging technique that offers cross-sectional images of the anterior segment to a depth of 5 mm (15). It can image and evaluate the morphological structure of the anterior segment of the eye, even the structure beyond the iris covering, such as the ciliary body, zonule, and peripheral and posterior lens without mydriasis (10). Moreover, UBM demonstrates high sensitivity, specificity, and accuracy for the detection of lens dislocation (15, 16).

In this study, patients with ASAC-LS presented with similar clinical characteristics and ocular features as observed in patients with APAC, namely female preponderance, worse BCVA, higher IOP, and shallower ACD than the unaffected eye. Moreover, in multiple studies involving the clinical and ocular features of APAC, the risk factors for APAC included old age, female sex, hyperopia, shallow ACD, thick and anterior placed lens, short AL, and ciliary body configurations (17–19). In this study, we demonstrated the same risk factors for patients with APAC. However, the patients with ASAC-LS had younger age, shallower ACD, and longer AL, compared with the patients with APAC. In addition, the tendency of ACD and AL in the ASAC-LS cohort was consistent with previous studies (8, 9).

An earlier study discovered that ACD is positively correlated with AL, in an aged population in South China (20). In the present study, however, the AL of both eyes within the ASAC-LS cohort was significantly longer than the APAC cohort. Moreover, the ACD of the unaffected eye of the ASAC-LS cohort was deeper than the APAC cohort, but ACD in the affected eye of the study groups was shallower than the control group. This discordance between the ACD and AL indirectly confirms that the lens position moves forward in the lens subluxation cohort, which may directly contribute to the shallower ACD in the ASAC-LS cohort, relative to the APAC cohort. Moreover, Kwon validated that the lens moves forward in the zonular instability patients, using indirect symptomology, such as less hyperopic spherical equivalent (SE), shallower ACD, and higher lens vault (LV) in the affected eyes (8).

The alteration of the lens position may be due to several pathological and/or physiological mechanisms. The zonular architecture is composed of three distinct regions. The anterior fibers can be traced from the anterior face of the lens, along with the depth of the valleys between the ciliary process, toward the ora serrata. The posterior fibers within the columns, on the other hand, extend from the posterior surface of the lens, past the ciliary processes near their apices toward the ora serrata. Finally, the intermediate fibers line the equator of the lens and remain between the anterior and posterior fibers in an intermediate position (21). From previous reports, lens subluxation is known to be related to an asymmetrical excessive laxity of the zonular fibers (13). Moreover, anterior zonules run a straight path from the ciliary body to the lens; therefore, any contraction or relaxation of the ciliary body directly reflects on the anterior lens capsule (22). Given these factors, we hypothesized that the weakness or dehiscence of the anterior zonules enables the lens to move forward. In addition, the weakness or dehiscence of the anterior zonules increases the curvature of the anterior lens capsule, thereby creating a shallower ACD, much like when the focusing distance of the accommodation. The anterior displaced lens increases iridolenticular contact, obturates pupil, and precludes the aqueous humor circulation, thus, forming a pupillary block. Consequently, the iris is pushed forward and the anterior chamber depth is decreased (8, 13).

Apart from ACD, we found that the ACD difference in the ASAC-LS cohort was bigger than the APAC cohort. In fact, the ACD difference in the ASAC-LS and APAC cohorts were 0.99 ± 0.52 mm and 0.15 ± 0.19 mm, respectively. Although past researchers reported a great difference in ACD in the center of each eye in patients with ASAC-LS, there were no reports on the exact difference between the two eyes of lens subluxation patients. To determine the risk factors for ASAC-LS, we performed logistic regression analysis and revealed that only the ACD difference was significantly associated with the lens subluxation. Since ACD is widely used for lens subluxation assessment in clinics, we next analyzed the ROC curve to assess the ASAC-LS diagnostic value of ACD and ACD difference. Based on our analysis, ACD < 1.4 mm and ACD difference > 0.63 mm are highly indicative of abnormal lens zonular dehiscence or relaxation. Unlike our study, Xing suggested that ACD < 1.25 mm reflected lens subluxation in their study (12). The difference in our studies may be due to the analysis of small sample sizes. Therefore, future investigations, involving a large cohort, are needed to gain better insight into the cut-off point of ACD and ACD difference in patients with ASAC-LS.

Our findings encountered several limitations. First, our patient population was relatively small. Hence, the conclusions, made in this study, are preliminary and require further confirmation with accumulative cases. Second, due to its retrospective design, participant information was collected *via* a review of medical records. At times, there was missing relevant information, such as medical history and medication use. Hence, further well designed, large-scale studies, such as measurement of central corneal thickness (CCT), lens thickness, and detailed measurement of the anterior chamber from UBM, are warranted.

In conclusion, in most cases of ASAC-LS, a definite cause can be established. This condition is primarily observed in patients with younger age, more frequent trauma history, lower IOP, longer AL, shallower ACD, and bigger ACD differences, relative to the patients with APAC. Based on our logistic regression analysis, ACD <1.4 mm and inter-eye ACD difference > 0.63 mm are highly indicative of lens subluxation. Our findings may offer new options in the diagnosis and differential diagnosis of ASAC-LS and APAC conditions.

## Data Availability Statement

The raw data supporting the conclusions of this article will be made available by the authors, without undue reservation.

## Ethics Statement

The studies involving human participants were reviewed and approved by Human Research Ethics Committee of the Eye and ENT Hospital of Fudan University. The patients/participants provided their written informed consent to participate in this study.

## Author Contributions

QJ was a major contributor in analyzing the data and writing the manuscript. TC contributed to the preparation of the tables and figures. ZC and LL collected the data. CZ provided critical revision. YJ was responsible for the research design. All authors contributed to the article and approved the submitted version.

## Funding

This study was funded by the National Natural Science Foundation of China (Grant No. 82070943) and the Shanghai Science and Technology Commission (Scientific Innovation Action Plan, Grant No. 20Y11911000).

## Conflict of Interest

The authors declare that the research was conducted in the absence of any commercial or financial relationships that could be construed as a potential conflict of interest.

## Publisher's Note

All claims expressed in this article are solely those of the authors and do not necessarily represent those of their affiliated organizations, or those of the publisher, the editors and the reviewers. Any product that may be evaluated in this article, or claim that may be made by its manufacturer, is not guaranteed or endorsed by the publisher.

## References

[1] Aboobakar IF Johnson WM Stamer WD Hauser MA Allingham RR. Major review: exfoliation syndrome; advances in disease genetics, molecular biology, and epidemiology. Exp Eye Res. (2017) 154:88–103. 10.1016/j.exer.2016.11.011 27845061

[2] Chu BS. Weill-Marchesani syndrome and secondary glaucoma associated with ectopia lentis. Clin Exp Optom. (2006) 89:95–9. 10.1111/j.1444-0938.2006.00014.x 16494613

[3] Hu R Wang X Wang Y Sun Y. Occult lens subluxation related to laser peripheral iridotomy. Medicine. (2017) 96:e6255. 10.1097/MD.0000000000006255 28272229 PMC5348177

[4] Jarrett WH. Dislocation of the lens. Archiv Ophthalmol. (1967) 78:289. 10.1001/archopht.1967.00980030291006 6040004

[5] Judge DP Dietz HC. Marfan's syndrome. Lancet. (2005) 366:1965–76. 10.1016/S0140-6736(05)67789-6 16325700 PMC1513064

[6] Ozdek S Bahceci UA Onol M Ezgu FS Hasanreisoglu B. Postoperative secondary glaucoma and anterior staphyloma in a patient with homocystinuria. J Pediatr Ophthalmol Strabismus. (2005) 42:243–6. 10.3928/01913913-20050701-09 16121557

[7] Sira M Ho T. Acute angle closure glaucoma secondary to a luxated lens associated with retinitis pigmentosa. Eye. (2005) 19:472–3. 10.1038/sj.eye.6701527 15184934

[8] Kwon J Sung KR. Factors associated with zonular instability during cataract surgery in eyes with acute angle closure attack. Am J Ophthalmol. (2017) 183:118–24. 10.1016/j.ajo.2017.09.003 28916480

[9] Luo L Li M Zhong Y Cheng B Liu X. Evaluation of secondary glaucoma associated with subluxated lens misdiagnosed as acute primary angle-closure glaucoma. J Glaucoma. (2013) 22:307–10. 10.1097/IJG.0b013e318241b85b 22218127

[10] Wang F Wang D Wang L. Characteristic manifestations regarding ultrasound biomicroscopy morphological data in the diagnosis of acute angle closure secondary to lens subluxation. Biomed Res Int. (2019) 2019:7472195. 10.1155/2019/7472195 31341905 PMC6614974

[11] Zhang Y Zong Y Jiang Y Jiang C Lu Y Zhu X. Clinical features and efficacy of lens surgery in patients with lens subluxation misdiagnosed as primary Angle-Closure glaucoma. Curr Eye Res. (2019) 44:393–8. 10.1080/02713683.2018.1548130 30426797

[12] Xing X Huang L Tian F Zhang Y Lv Y Liu W. Biometric indicators of eyes with occult lens subluxation inducing secondary acute angle closure. BMC Ophthalmol. (2020) 20:87. 10.1186/s12886-020-01355-7 32138781 PMC7059282

[13] Dureau P. Pathophysiology of zonular diseases. Curr Opin Ophthalmol. (2008) 19:27–30. 10.1097/ICU.0b013e3282f29f01 18090894

[14] Wan T Yin H Yang Y Wu F Wu Z Yang Y. Comparative study of anterior segment measurements using 3 different instruments in myopic patients after ICL implantation. BMC Ophthalmol. (2019) 19:182. 10.1186/s12886-019-1194-y 31409385 PMC6693247

[15] Shi M Ma L Zhang J Yan Q. Role of 25 MHz ultrasound biomicroscopy in the detection of subluxated lenses. J Ophthalmol. (2018) 2018:3760280. 10.1155/2018/3760280 30416825 PMC6207873

[16] Ojaghi HS Morteza BH Sorkhabi R Tarzamani MK Kamali ZG Mikaeilpour A. Diagnostic accuracy of ultrasound in detection of traumatic lens dislocation. Emerg (Tehran). (2014) 2:121–4. 26495362 PMC4614573

[17] Gedde SJ Chen PP Muir KW Vinod K Lind JT Wright MM. Primary angle-closure disease preferred practice pattern(R). Ophthalmology. (2021) 128:P30–70. 10.1016/j.ophtha.2020.10.021 34933744

[18] Sun X Dai Y Chen Y Yu D Cringle SJ Chen J. Primary angle closure glaucoma: what we know and what we don't know. Prog Retin Eye Res. (2017) 57:26–45. 10.1016/j.preteyeres.2016.12.003 28039061

[19] Wright C Tawfik MA Waisbourd M Katz LJ. Primary angle-closure glaucoma: an update. Acta Ophthalmol. (2016) 94:217–25. 10.1111/aos.12784 26119516

[20] Chen H Lin H Lin Z Chen J Chen W. Distribution of axial length, anterior chamber depth, and corneal curvature in an aged population in South China. BMC Ophthalmol. (2016) 16:47. 10.1186/s12886-016-0221-5 27138378 PMC4852406

[21] Mcculloch C. The zonule of Zinn: Its origin, course, and insertion, and its relation to neighboring structures. Trans Am Ophthalmol Soc. (1954) 52:525–85. 13274438 PMC1312608

[22] Bernal A Parel J Manns F. Evidence for posterior zonular fiber attachment on the anterior hyaloid membrane. Investigative Opthalmology and Visual Science. (2006) 47:4708. 10.1167/iovs.06-0441 17065477

